# Catheter ablation versus medical therapy in atrial fibrillation: an umbrella review of meta-analyses of randomized clinical trials

**DOI:** 10.1186/s12872-023-03670-5

**Published:** 2024-02-29

**Authors:** Anoop Titus, Sakil Syeed, Abiram Baburaj, Karan Bhanushali, Pramod Gaikwad, Mannil Sooraj, Anu Mariam Saji, Wasey Ali Yadullahi Mir, Pramukh Arun Kumar, Mahati Dasari, Mubashir Ayaz Ahmed, Mohammed Omer Khan, Aishwarya Titus, Janamjey Gaur, Dilanthy Annappah, Arjun Raj, Nabeela Noreen, Adrian Hasdianda, Yasar Sattar, Bharat Narasimhan, Nishaki Mehta, Christopher V. Desimone, Abhishek Deshmukh, Sarju Ganatra, Khurram Nasir, Sourbha Dani

**Affiliations:** 1 DeBakey Heart and Vascular Center, Houston Methodist, Houston, TX USA; 2 University of Utah, Salt Lake City, UT USA; 3 Queen’s University Belfast, Belfast, UK; 4 Roger Williams Medical Center, Providence, RI USA; 5 Massachusetts General Hospital, Boston, MA USA; 6 Dr. Chandramma Dayananda Sagar Institute of Medical Education and Research, Kanakapura, Karnataka India; 7 Saint Vincent Hospital, Worcester, MA USA; 8 Saint Elizabeth Medical Center, Boston, MA USA; 9 Ascension Saint Joseph Hospital, Chicago, IL USA; 10 Rush University Medical Center, Chicago, IL USA; 11 Pushpagiri Institute of Medical Sciences and Research Centre, Thiruvalla, Kerala India; 12 Canton Medical Education Foundation, Canton, OH USA; 13 University Hospital of Leicester, Leicester, UK; 14 Rajshahi Medical College, Rajshahi, Bangladesh; 15 Brigham and Women’s Hospital, Harvard University, Cambridge, MA USA; 16 West Virginia University, Morgantown, WV USA; 17 Beaumont Hospital Royal Oak, Oakland University William Beaumont School of Medicine, Royal Oak, MI USA; 18 Mayo Clinic, Rochester, Minnesota USA; 19 Department of Cardiology, Lahey Hospital and Medical Center, Beth Israel Lahey Health, 41 Mall Road, Burlington, MA 10805 USA

**Keywords:** Atrial fibrillation, Catheter ablation, Medical treatment, Meta-analysis, Mortality, Cardiovascular hospitalization, Heart failure, Pulmonary vein stenosis, Left ventricular ejection fraction, Major bleeding

## Abstract

This umbrella review synthesizes data from 17 meta-analyses investigating the comparative outcomes of catheter ablation (CA) and medical treatment (MT) for atrial fibrillation (AF). Outcomes assessed were mortality, risk of hospitalization, AF recurrence, cardiovascular events, pulmonary vein stenosis, major bleeding, and changes in left ventricular ejection fraction (LVEF) and MLHFQ score. The findings indicate that CA significantly reduces overall mortality and cardiovascular hospitalization with high strength of evidence. The risk of AF recurrence was notably lower with CA, with moderate strength of evidence. Two associations reported an increased risk of pulmonary vein stenosis and major bleeding with CA, supported by high strength of evidence. Improved LVEF and a positive change in MLHFQ were also associated with CA. Among patients with AF and heart failure, CA appears superior to MT for reducing mortality, improving LVEF, and reducing cardiovascular rehospitalizations. In nonspecific populations, CA reduced mortality and improved LVEF but had higher complication rates. Our findings suggest that CA might offer significant benefits in managing AF, particularly in patients with heart failure. However, the risk of complications, including pulmonary vein stenosis and major bleeding, is notable. Further research in understudied populations may help refine these conclusions.

**Supplementary Information:**

The online version contains supplementary material available at 10.1186/s12872-023-03670-5.

## Introduction

Atrial fibrillation (AF) is a chronic and progressive medical condition associated with substantial morbidity, functional and quality of life (QoL) impairment, and increased mortality risk [1, 2]. … When symptomatic, AF negatively impacts the quality of life (QoL) due to accompanying cardiac or noncardiac symptoms such as palpitations, shortness of breath, and extreme fatigue [3]. Furthermore, as the population ages, AF becomes more prevalent and poses increased risks of morbidity and mortality from AF-related complications, primarily stroke and congestive heart failure [4].

Since its introduction in 1998, catheter ablation (CA) has been an effective rhythm-control strategy for symptomatic AF patients [5, 6]. CA has made continuous progress, resulting in improved procedural success and complication rates [7]. However, AF management still relies heavily on medical treatment (MT) [8]. Medical treatment is limited in certain conditions due to lack of efficacy, proarrhythmic effects, limited use in patients with kidney or liver disease, and drug‒drug interactions [8]. The effect of ablation on patients with heart failure has reduced cardiovascular rehospitalization and AF recurrence.

Several studies have compared the outcomes of CA vs. MT for AF. For example, the CABANA (Catheter Ablation vs. Antiarrhythmic Drug Therapy for Atrial Fibrillation) trial reported that CA did not significantly reduce the primary end point of death, disabling stroke, severe bleeding, or cardiac arrest compared with MT [9]. Several meta-analyses comparing CA vs. MT reported outcomes such as a reduction in all-cause mortality and hospitalizations, improvement in left ventricular ejection fraction (LVEF), and greater freedom from atrial arrhythmia and AF [10]. However, contrasting evidence on the effectiveness of CAs for these outcomes is available from meta-analyses, and the determination of the credibility of these findings remains to be assessed. Umbrella reviews provide a structured and critical summary of the evidence from several meta-analyses and enable the grading of evidence by evaluating the strength and precision of the associations and the presence of bias [11, 12].

In this umbrella review, we aimed to systematically identify relevant meta-analyses of RCTs, summarize their findings, and assess the evidence to provide a comprehensive picture of the outcomes of CAs vs. MT.

## Methods

The protocol for this study was registered with the Open Science Framework (OSF) (https://osf.io/v436d). This umbrella review was reported following the 2020 Preferred Reporting Items for Systematic Reviews and Meta-analyses (PRISMA) reporting guidelines [13].

### Search strategy and eligibility criteria

We searched PubMed, EMBASE, Epistemonikos, and the Cochrane Database of Systematic Reviews (CDSR) from database inception to Feb 28, 2023. No language restriction was applied. Search results were imported to EndNote20, and duplicate references were removed. The library was then exported to Rayyan.ai for title and abstract screening. Each article was screened by the title and abstract by two randomly selected authors (P. G, A. S, W. M, P. K, M. D, M. A, M. K, Ai. T, J. G, A. R, N. N) to check for eligibility for the study. Randomization of authors and blinding was performed with Rayyan.ai. Any discrepancies were resolved by discussion with the third reviewer (S.S.).

**Fig. 1:**
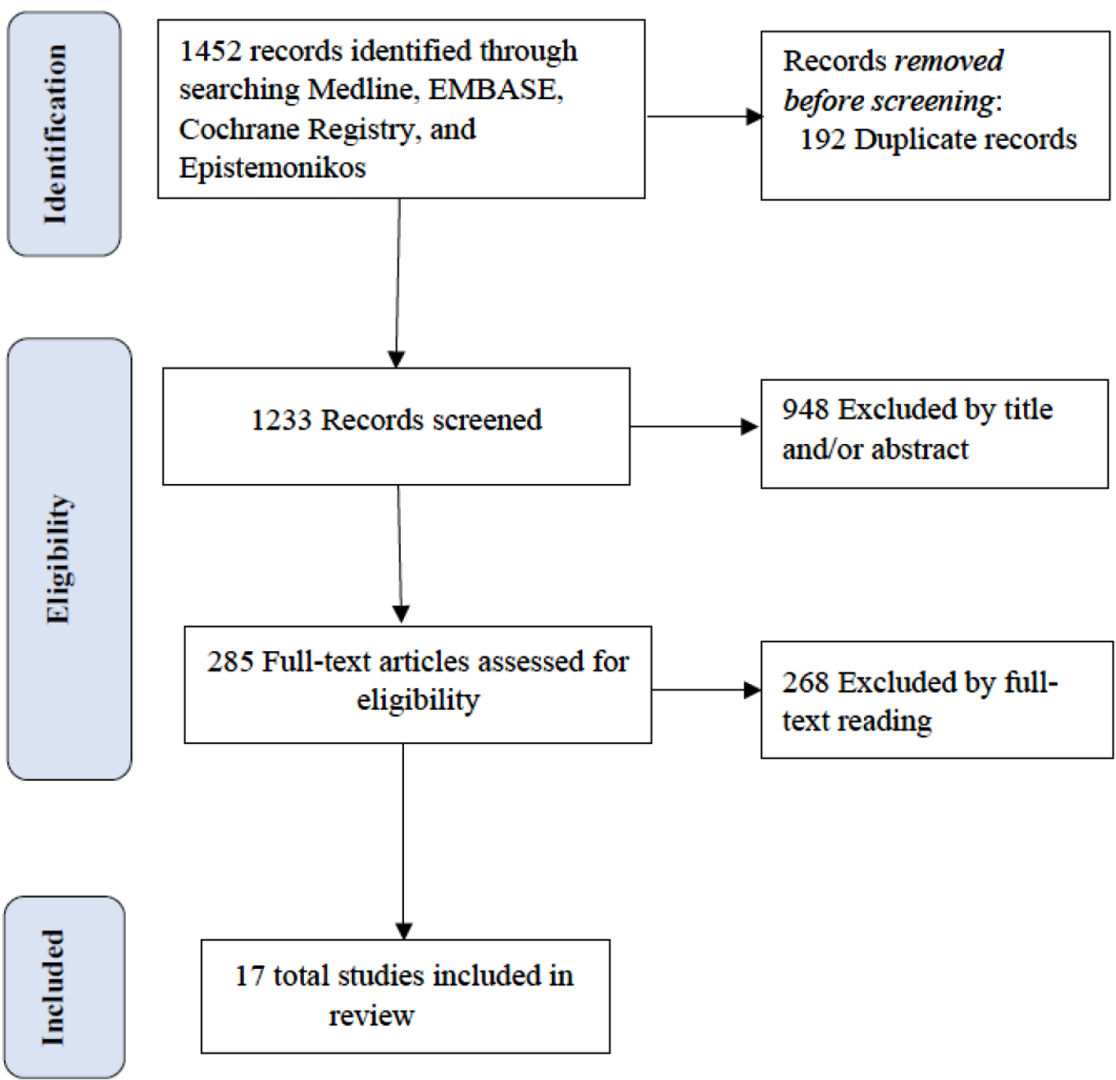
PRISMA flow diagram

We included meta-analyses of RCTs investigating the outcomes of AF, comparing CA with MT. When more than one meta-analysis study was available for the same research question, we selected the meta-analysis with the most extensive data set, as previously described [14–16]. We excluded (i) meta-analyses of studies with other study designs (e.g., cohort, case–control studies); (ii) pooled analyses of a nonsystematic selection of observational studies and nonsystematic reviews; and (iii) meta-analyses that provided insufficient or inadequate data for quantitative synthesis. Subgroup categories of studies included (a) patients with heart failure (HF), (b) patients without HF, (c) patients with and without HF, and (d) patients with unspecified HF. The detailed PRISMA flow diagram is as shown in Fig. 1.

### Data extraction and quality assessment

Two reviewers (MS, AB) independently performed data extraction and quality assessment, which was checked by another two reviewers (SS, AT). Any existing discrepancies were resolved by consensus. The quality of meta-analyses was assessed using the AMSTAR-2-A Measurement Tool to Assess Systematic Reviews and graded as high, moderate, low, or critically low [17]. Sheets of the extracted data are available online on the Mendeley Data repository.

### Statistical analysis

We extracted effect sizes from individual studies that were categorized based on the population, intervention, comparator, and outcomes to generate the unique association in AF patients receiving CA vs. MT. For each association, effect sizes (mean difference [MD], standardized mean difference [SMD], and risk ratio [RR]) of individual studies were extracted. Then, the meta-analyses were reperformed to calculate the pooled effect sizes and 95% CIs using a random-effects model under the DerSimonian and Laird method [21]. The I^2^ statistic was utilized to assess heterogeneity [18]. Egger regression asymmetry was used to determine the evidence of small-study effects [19]. A P value of < 0.10 was taken as statistical evidence for the presence of small-study effects. An I^2^ value ≥ 70% was considered significant heterogeneity. Statistical analyses were conducted using Stata version 17.0 (StataCorp, College Station, TX, USA).

### Assessment of the credibility of the evidence

We evaluated the quality of evidence per association provided in a meta-analysis of RCTs using the GRADE criteria (Grading of Recommendations, Assessment, Development, and Evaluations) framework to classify the evidence as high, moderate, low, and very low [20]. Five domains, including (1) risk of bias in the individual studies, (2) inconsistency, (3) indirectness, (4) imprecision, and (5) publication bias, were assessed using GRADEpro version 3.6.1 (McMaster University) to generate the credibility of the evidence.

## Results

### Cohort characteristics

We identified 1,452 studies, scrutinized 252 full-text articles, and ultimately included 17 meta-analyses in this umbrella review. Seventeen eligible studies [21–36] described 33 potential associations/meta-analyses, including 33 individual meta-analyses of outcomes associated with CA. The descriptive characteristics of the included studies are provided in Supplementary Table 1. The median number of RCTs per meta-analysis was 8.5 (interquartile interval [IQI]: 6.75-11), with a median sample size of 2,496 (IQI: 1,038 − 3,714).

### Study associations and strength of evidence

A summary of all 33 associations is presented in Supplementary Table 1. Twenty-one of the 33 examined associations (63.6%) were statistically significant at p < 0.05. Seventeen associations (51.2%) had considerable heterogeneity.

The strength of evidence assessed using GRADE found that 30 had equivalent levels of support from high, moderate, and low strength of evidence (10 associations each, [30.3%]). In the rest of the comparison, three associations were supported by deficient levels of evidence (0.1%).

### Study outcomes

#### Mortality

Among the 21 statistically significant associations (Supplementary Table 1), two reported a decreased overall mortality risk (RR, 0.55 to 0.72) with CA compared to MT. They were supported by a high strength of evidence.

#### Risk of hospitalization

Of the three associations that reported a significantly lowered risk of cardiovascular hospitalization, one association [30] was supported by high strength of evidence (RR, 0.37).

#### Risk of AF recurrence

One significant association reported a reduced risk of AF recurrence (RR, 0.46) with CA, backed by moderate strength of evidence.

#### Risk of cardiovascular events and arrhythmias

Two other associations of reduced risk of a cardiovascular event and recurring atrial arrhythmia were reported with moderate and low strength of evidence, respectively.

#### Risk of pulmonary vein stenosis

One significant associations reported an increased risk of pulmonary vein stenosis (RR, 2.34) with high strength of evidence with CA.

#### Major bleeding

One significant associations reported an increased risk of major bleeding (RR, 3.88) with high strength of evidence with CA.

#### Change in LVEF and MLHFQ

**Fig. 2:**
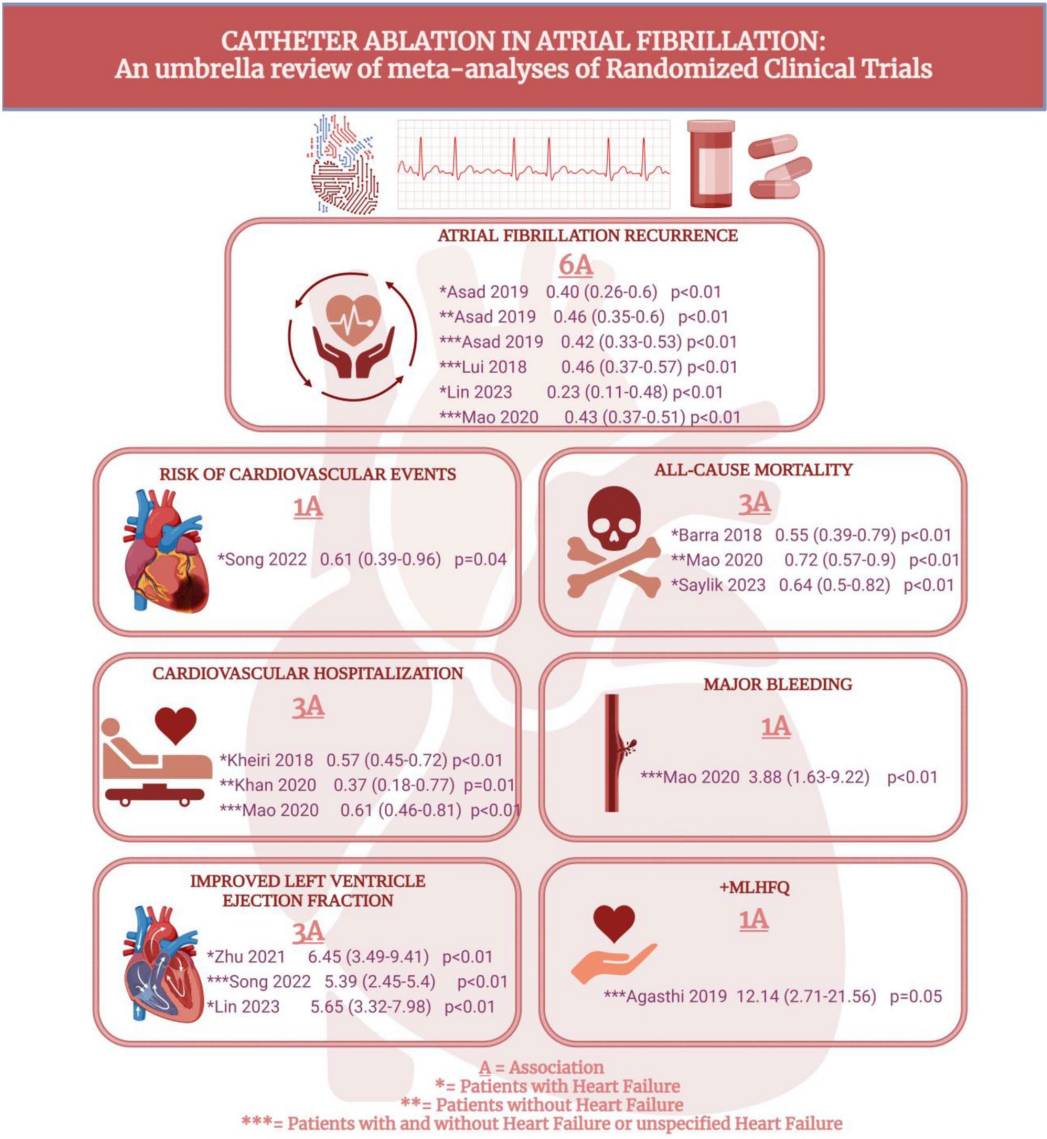
Central illustration visual representation of findings from the umbrella review

Two associations at moderate and low strength of evidence reported an improved LVEF (MD, 5.65–6.45), and one at high strength of evidence reported a positive change in the ‘Minnesota Living with heart failure questionnaire,’ (MLHFQ) (MD, 12.14) from CA. The summary of the outcomes is as shown in Fig. 2.

## Discussion

In this umbrella review, we summarize 33 studies with clinical outcomes in four broad atrial fibrillation (AF) patient categories: (a) patients with heart failure (HF), (b) patients without HF, (c) patients with and without HF, and (d) patients with unspecified HF. Our analysis helps broaden our understanding of the patient population with AF that would benefit the most from a CA.

The main findings of this study are as follows:

1. CA is superior to MT in reducing mortality in a patient population with HF (high certainty) and a population with and without HF (moderate certainty).
2. The improvement in LVEF was greater in the CA group than in the MT group in patients with HF (very low certainty) and patients with unspecified HF (low certainty).
3. CA is better than MT in cardiovascular hospitalization rates in most groups, except for patients with unspecified HF. However, the supported evidence certainty is high only in the HF group.
4. The AF recurrence rate was better in all groups treated with CA with low certainty of evidence.

Complications, including major bleeding and pulmonary vein stenosis, are high in the CA arm in patients with and without HF supported with high certainty. The overall incidence of peri-ablation complications is low.

### All-cause mortality

Our analysis shows that CA was superior to MT in mortality outcomes in two AF groups: (a) patients with HF, as studied by Barra et al. [30] and (b) patients with and without HF, as studied by Mao et al. [29]. The associated certainty of the evidence is high to moderate, and the quality of meta-analyses reporting these results is high. There was no heterogeneity found in the results from both meta-analyses. AF per se is associated with the risk of morbidity and mortality, but when it occurs in conjunction with HF, it is associated with increased mortality [37, 38].

Patients with heart failure with reduced ejection fraction (HFrEF) depend on atrial contraction to maintain sound cardiac output, and treating AF also reduces the incidence of tachycardia-mediated cardiomyopathy [39]. Multiple studies have demonstrated that ablation is superior to antiarrhythmics in maintaining sinus rhythm [40, 41]. This effect on improved left ventricular function is probably associated with the mortality benefit in the CA arm [42, 43]. Barra et al. reported on the AATAC and CASTLE AF trials [30]One explanation for why Mao et al. failed to demonstrate mortality benefits in the CA group in patients without HF is due to low event rates [29]. It is estimated that more than 6000 patients need to be recruited in the non-HF arm [44]. The authors reported that the CA arm had mortality benefits in the group, including patients with and without HF. This finding was linked to the HF studies included in the analysis. A study by Zheng et al. showed improved all-cause mortality in the CA arm in patients with unspecified HF [28]. However, the results were not statistically significant (p = 0.745), and the quality of the meta-analysis on AMSTAR 2 was critically low. Multiple small studies with a shorter follow-up duration (< 1 year) are a significant drawback of the Zheng et al. analysis. Data on mortality outcomes in patients with heart failure with preserved ejection fraction are limited to a few observational studies [45–51]. In a retrospective study by Hayrioglu MI et al. on octogenerians implanted with dual chamber permanent pacemakers, the presence of AF was found to be an independent risk factor for long-term mortality [52]. Our current analysis shows that CA is a successful strategy for improving hard endpoints such as mortality.

### Improvement in LVEF

Atrial fibrillation can lead to worsening of HF by the following mechanisms: a) AF-induced loss of atrial’systole’ impairs the filling of the left ventricle (LV) during ventricular diastole, which causes the cardiac output to drop by up to 25%, and b) irregular and rapid ventricular conduction can cause tachycardia-induced cardiomyopathy. Restoration of sinus rhythm improves cardiac function by increasing stroke volume and LV emptying even before the improvement of LV contractility [53]. The CAMERA-MRI study demonstrated that restoration of sinus rhythm in the CA arm causes improvement in left ventricular function, especially in the absence of ventricular fibrosis on cardiac MRI [54]. This highlights the importance of restoring sinus rhythm in improving LVEF. Zhu et al. reported improvement in LVEF in the CA arm compared to MT in patients with HF with very low certainty of evidence [27]. In a different meta-analysis, Song et al. reported similar findings in patients with unspecified HF with low certainty [26]. They also performed a trial sequel analysis of the LVEF outcome, improving precision. However, both studies reported that their outcomes have high heterogeneity. This difference could be explained by the different cardiac imaging modalities used in various studies and the observer-dependent variation associated with measuring LVEF [55].

### All cardiovascular events and stroke

In the current umbrella review, Song et al. is the only study that shows a lower rate of cardiovascular events in the CA arm, seen in patients with HF. The strength of evidence is moderate and low quality on AMSTAR2 evaluation. This study included 11 studies analyzing stroke outcomes, which is a notable strength of this study. The CAPA trial is the only trial that has shown statistically significant stroke reduction [56]. The population included in this trial was younger, with a lower CHA2DS2VASc score than that seen in the CABANA trial. Other meta-analyses failed to demonstrate statistically significant results regarding stroke events, as they needed more power [25, 29]. The CABANA trial, even though being the largest of the studies, failed to show positive outcomes for the following reasons: a) reduced ‘true’ estimate effect due to high rate of crossovers and withdrawals, affecting the results of intention to treat analysis and b) background anticoagulation causing a lesser number of strokes in both arms [9]. This UA demonstrates that CA is safe for preventing CV events; however, more robust evidence is warranted. Current guidelines recommend anticoagulation after ablation beyond two months based on the individual patient’s risk profile, which is the CHA2-DS2VASc score (Class IC-EO) [57]. The OCEAN trial is an ongoing study evaluating optimal antithrombotic treatment strategies for patients with stroke risk factors after successful ablation [58].

### Change in MLHFQ and cardiovascular hospitalization

Our analysis shows that CA, compared to MT, showed the most significant improvement in the MLHFQ; however, there was high heterogeneity [23]. This effect is seen in patients with unspecified HF, and the quality of evidence, according to the GRADE system, is low. Other studies by Zhu et al. (low certainty on GRADE) in patients with HF and Shi et al. (very low certainty on GRADE) in patients with and without HF, respectively, also showed improvement in MLHFQ, both studies with heterogeneity [24, 27]. Except for the ATTAC trial, all other studies included in the analyses were small and had a short follow-up duration.

Previous studies have shown that the time spent in sinus rhythm is associated with improved quality of life [59]. The results from the ATTAC trial also show that CA is superior to amiodarone in maintaining sinus rhythm and increased exercise tolerance in patients with AF and HFrEF [43]. Health care costs significantly influence quality of life (QOL). Multiple studies show that CA is cost-effective in the long term, even though it has a high upfront cost [60, 61]. However, there are some challenges with studying the actual effect of CA on QOL that are worth mentioning. Blinding quality assessment is very difficult, and additionally, there is a risk of bias due to the subjective nature of QOL assessment.

### Cardiovascular hospitalization

The rate of cardiovascular (CV) hospitalization was lower in the CA arm than in the MT arm in three patient groups: (a) patients with HF, (b) patients without HF, and (c) patients with and without HF. All three results are statistically significant, with only Kheiri et al. showing no heterogeneity in their analysis. The certainty of the evidence is high in the HF group (Kheiri et al.) and low in the non-HF group as well as with and without the HF group (Khan et al., Mao et al.). Mao et al. is the only high-quality analysis on AMSTAR 2 grading. A retrospective cohort study of the nationwide readmissions database (NRD) by Arora et al. showed that at one year, CA reduced readmissions due to AF but did not reduce readmissions due to heart failure, irrespective of the type of HF [62]. However, given the study’s observational nature and NRD being an administrative database, one should be cautious when interpreting these results.

### Periprocedural complications

#### Major bleeding

In this analysis, major bleeding complications due to CA were reported to be high in the study by Mao et al. [29]. Zheng et al. noted a high rate of cardiac tamponade in CA vs. AADs, but the absolute incidence was 0.55%. A large meta-analysis by Gupta et al. that included > 80,000 patients undergoing CA showed a low incidence of periprocedural complications. The complication rate has declined over the years, reflecting improved ablation technology and experience worldwide [63]. Real-world data from an extensive United States hospital database also show low complication rates [64]. These complications may be affected by the patient’s comorbidities, ablation strategy, experience of the operator, and adequacy of periprocedural anticoagulation. Our analysis showed that CA is a relatively safe procedure with a small risk of periprocedural complications.

#### Pulmonary vein stenosis

Mao et al. [29] reported higher rates of pulmonary vein stenosis associated with CA. Early ablative techniques, which targated focal ablation directly within the venous ostia have demonstrated an increased incidence of pulmonary vein stenosis. Improved understanding of risk factors for pulmonary vein stenosis and adoption of newer ablation techniques like circumferential ablation and antral isolation have led to substantial reduction in pulmonary vein stenosis [65].

### AF recurrence

Overall, studies on AF recurrence resulted in CA’s superiority over MT. Asad et al. reported positive results in patients with HF, patients without HF, and patients with/without HF, all three outcomes with high heterogeneity [22]. The strength of evidence is low, and the quality of the meta-analyses is moderate. Lui et al. reported similar results in patients with unspecified HF, with low certainty of evidence [21]. Asad et al. showed that CA was superior to MT in paroxysmal and persistent AF.

Similarly, Lui et al. showed it as an effective first-line and second-line therapy strategy. Most clinical trials focused on AF recurrence as an outcome in a binary fashion. However, a more relevant parameter would be the assessment of the overall AF burden measured as percent time in AF, number of episodes, and duration of the most extended episode [66]. The ARREST-AF study is a trial that should show that aggressive risk factor reduction improves the long-term success of AF ablation [67]. This strategy could positively impact other AF outcomes and needs to be validated in larger RCTs. Pulmonary vein isolation (PVI) continues to remain the cornerstone of CA for AF treatment. Most of the times PVI is carried out empirically based on the hypothesis that the pulmonary veins are the source of ecopic trigger. However, AF can be trigger by many non PV related sources, causing recurrence of AF after a successful PVI. Several ablation strategies in addition to pulmonary vein isolation (PVI) including left atrial posterior wall isolation, superior venacava isolation, ligament of Marshall and coronary sinus ablation, ganglion plexus ablation and renal denervation have been tried in managing AF recurrence. The results from these strategies have been very heterogenous and future studies are needed to determine the appropriate strategy to manage AF recurrence [68].

### Clinical implication and future direction

Numerous prediction risk score models help determine an intervention’s outcomes. For example, the ATLAS score is a clinical tool used to estimate the rate of AF recurrence after a CA procedure. Our umbrella review can help build a similar model that would help predict the patient population that would greatly benefit from CA. Future trials should focus on a patient population that is not well studied, for example, patients with HFpEF, females, and the elderly population. In the last decade, artificial intelligence (AI) has shown its effectiveness in AF management in various ways. AI- enabled electrocardiogram (ECG) algorithm was found to be effective in predicting the recurrence of paroxysmal AF following CA.The future of AI guided AF management is promising, however currently the high-quality data required to develop AI systems in still limited [52].

### Strengths and limitations

This umbrella analysis is the largest of its kind, studying 28 outcomes simultaneously associated with CA for atrial fibrillation. For each outcome, the meta-analysis that included the most significant number of studies was included in the review, thus providing adequate statistical power. Each association was qualitatively assessed on the GRADE scale for strength of evidence, and all the meta-analyses included in the current review were analyzed using AMSTAR2 to evaluate their quality.

Despite these advantages, there were some limitations in our present study. We did not appraise the quality of primary studies included in the meta-analyses. Some primary studies included in the meta-analyses were smaller with short follow-ups, and some were open-labeled, which can potentiate bias. Some notable methodological heterogeneity relates to the preliminary study’s different ablation techniques, AF recurrence detection methods, single vs. multiple ablations, and anticoagulation protocols. A few patient-level factors, including the type of AF (paroxysmal vs. persistent AF), degree of HF, and patients’ CHA2DS2VASC, could affect clinical estimates but were not studied in this current UA.

## Conclusion

This umbrella review suggests that in patients with AF and heart failure, CA is superior to MT for reducing mortality (with high certainty), improving LVEF (very low certainty), and reducing cardiovascular rehospitalizations (high certainty). In a nonspecific population study comprising patients known to be with and without heart failure, CA is seen to be superior to MT for reducing mortality (with moderate certainty), improving LVEF (low certainty), and increasing complication rates such as pulmonary vein stenosis and major bleeding events (high certainty). In studies with populations with an unknown history of heart failure, overall, CA was a safe procedure with a small risk of periprocedural complications.

### Electronic supplementary material

Below is the link to the electronic supplementary material.

**Supplementary Material 1:** Summary of studies

**Supplementary Material 2:** AMSTAR 2: Critical appraisal tool for systematic reviews that includes randomized or non-randomized studies of healthcare interventions, or both

## Data Availability

The datasets used/analysed during the current study are available from the corresponding author on reasonable request.

## References

[1] Lippi G, Sanchis-Gomar F, Cervellin G (2021). Global epidemiology of atrial fibrillation: an increasing epidemic and public health challenge. Int J Stroke.

[2] Nattel S (2002). New ideas about atrial fibrillation 50 years on. Nature.

[3] Rienstra M, Lubitz SA, Mahida S, Magnani JW, Fontes JD, Sinner MF (2012). Symptoms and functional status of patients with atrial fibrillation: state of the art and future research opportunities. Circulation.

[4] Kornej J, Börschel CS, Benjamin EJ, Schnabel RB (2020). Epidemiology of Atrial Fibrillation in the 21st Century: Novel methods and New insights. Circ Res.

[5] Parameswaran R, Al-Kaisey AM, Kalman JM (2021). Catheter ablation for atrial fibrillation: current indications and evolving technologies. Nat Rev Cardiol.

[6] Haïssaguerre M, Jaïs P, Shah DC, Takahashi A, Hocini M, Quiniou G (1998). Spontaneous initiation of atrial fibrillation by ectopic beats originating in the pulmonary veins. N Engl J Med.

[7] Kalman JM, Sanders P, Rosso R, Calkins H (2017). Should we perform catheter ablation for asymptomatic Atrial Fibrillation?. Circulation.

[8] Zimetbaum P (2012). Antiarrhythmic drug therapy for atrial fibrillation. Circulation.

[9] Packer DL, Mark DB, Robb RA, Monahan KH, Bahnson TD, Poole JE (2019). Effect of catheter ablation vs antiarrhythmic drug therapy on mortality, Stroke, bleeding, and Cardiac Arrest among patients with Atrial Fibrillation. JAMA.

[10] Ravi V, Poudyal A, Lin L, Larsen T, Wasserlauf J, Trohman RG (2022). Mortality benefit of catheter ablation versus medical therapy in atrial fibrillation: an RCT only meta-analysis. J Cardiovasc Electrophysiol.

[11] Ioannidis JPA (2009). Integration of evidence from multiple meta-analyses: a primer on umbrella reviews, treatment networks and multiple treatments meta-analyses. CMAJ.

[12] Ioannidis J (2017). Next-generation systematic reviews: prospective meta-analysis, individual-level data, networks and umbrella reviews. Br J Sports Med.

[13] Page MJ, McKenzie JE, Bossuyt PM, Boutron I, Hoffmann TC, Mulrow CD (2021). The PRISMA 2020 statement: an updated guideline for reporting systematic reviews. Syst Rev.

[14] Dragioti E, Solmi M, Favaro A, Fusar-Poli P, Dazzan P, Thompson T (2019). Association of antidepressant use with adverse Health outcomes: a systematic Umbrella Review. JAMA Psychiatry.

[15] Neuenschwander M, Ballon A, Weber KS, Norat T, Aune D, Schwingshackl L (2019). Role of diet in type 2 Diabetes incidence: umbrella review of meta-analyses of prospective observational studies. BMJ.

[16] Veettil SK, Wong TY, Loo YS, Playdon MC, Lai NM, Giovannucci EL (2021). Role of Diet in Colorectal Cancer incidence: Umbrella Review of Meta-analyses of prospective observational studies. JAMA Netw Open.

[17] Shea BJ, Reeves BC, Wells G, Thuku M, Hamel C, Moran J et al. AMSTAR 2: a critical appraisal tool for systematic reviews that include randomised or non-randomised studies of healthcare interventions, or both. BMJ. 2017;j4008. 10.1136/bmj.j4008 PMC5833365 28935701

[18] Riley RD, Higgins JPT, Deeks JJ (2011). Interpretation of random effects meta-analyses. BMJ.

[19] Stuck AE, Rubenstein LZ, Wieland D (1998). Bias in meta-analysis detected by a simple, graphical test. Asymmetry detected in funnel plot was probably due to true heterogeneity. BMJ.

[20] Langendam MW, Akl EA, Dahm P, Glasziou P, Guyatt G, Schünemann HJ (2013). Assessing and presenting summaries of evidence in Cochrane Reviews. Syst Rev.

[21] Liu W, Wu Q, Yang XJ, Huang J (2018). The trend of change in catheter ablation versus antiarrhythmic Drugs for the management of atrial fibrillation over time: a meta-analysis and meta-regression. J Geriatr Cardiol.

[22] Asad ZUA, Yousif A, Khan MS, Al-Khatib SM, Stavrakis S. Catheter ablation Versus Medical Therapy for Atrial Fibrillation. Circ Arrhythm Electrophysiol. 2019;12(9). 10.1161/CIRCEP.119.007414 31431051

[23] Agasthi P, Lee JZ, Amin M, Al-Saffar F, Goel V, Tseng A (2019). Catheter ablation for treatment of atrial fibrillation in patients with Heart Failure with reduced ejection fraction: a systematic review and meta‐analysis. J Arrhythm.

[24] SHI LZ, HENG R, LIU SM, LENG FY (2015). Effect of catheter ablation versus antiarrhythmic Drugs on atrial fibrillation: a meta-analysis of randomized controlled trials. Exp Ther Med.

[25] Muhammad ZK, Safi UK, Adeel A, Muhammad SZ, Muhammad UK, Muhammad SK (2020). Meta-analysis of catheter ablation versus medical therapy in patients with Atrial Fibrillation without Heart Failure. J Atr Fibrillation.

[26] Song J, Zhang Q, Ye L, Zheng Y, Wang L (2022). The comparison of catheter ablation on hard outcomes versus medical treatment for atrial fibrillation patients: a meta-analysis of randomized, controlled trials with trial sequential analysis. PLoS ONE.

[27] Zhu X, Wu Y, Ning Z (2021). Meta-analysis of catheter ablation versus medical therapy for Heart Failure complicated with Atrial Fibrillation. Cardiol Res Pract.

[28] Zheng ZH, Fan J, Ji CC, Cheng YJ, Chen XM, Jiang JZ (2021). Long-term outcomes and improvements in quality of life in patients with Atrial Fibrillation Treated with catheter ablation vs. Antiarrhythm Drugs Am J Cardiovasc Drugs.

[29] Mao Y jun, Wang H, Chen J, xing, Huang P. fang. Meta-analysis of medical management versus catheter ablation for atrial fibrillation. Rev Cardiovasc Med. 2020;21(3):419. 10.31083/j.rcm.2020.03.60 33070546

[30] Barra S, Baran J, Narayanan K, Boveda S, Fynn S, Heck P (2018). Association of catheter ablation for atrial fibrillation with mortality and Stroke: a systematic review and meta-analysis. Int J Cardiol.

[31] Androulakis E, Sohrabi C, Briasoulis A, Bakogiannis C, Saberwal B, Siasos G (2022). Catheter ablation for Atrial Fibrillation in patients with Heart Failure with preserved ejection fraction: a systematic review and Meta-analysis. J Clin Med.

[32] Kheiri B, Osman M, Abdalla A, Haykal T, Ahmed S, Bachuwa G (2018). Catheter ablation of atrial fibrillation with Heart Failure: an updated meta-analysis of randomized trials. Int J Cardiol.

[33] Yi F, Hou W, Zhou C, Yin Y, Lu S, Duan C (2019). Radiofrequency ablation Versus Antiarrhythmic Drug Therapy for Atrial Fibrillation: Meta-analysis of Safety and Efficacy. J Cardiovasc Pharmacol.

[34] Chen S, Pürerfellner H, Meyer C, Acou WJ, Schratter A, Ling Z (2020). Rhythm control for patients with atrial fibrillation complicated with Heart Failure in the contemporary era of catheter ablation: a stratified pooled analysis of randomized data. Eur Heart J.

[35] Lin C, Sun M, Liu Y, Su Y, Liang X, Ma S et al. Catheter ablation vs. drug therapy in the treatment of atrial fibrillation patients with Heart Failure: an update meta-analysis for randomized controlled trials. Front Cardiovasc Med. 2023;10. 10.3389/fcvm.2023.1103567 PMC10031055 36970339

[36] Şaylık F, Çınar T, Akbulut T, Hayıroğlu Mİ (2023). Comparison of catheter ablation and medical therapy for atrial fibrillation in Heart Failure patients: a meta-analysis of randomized controlled trials. Heart & Lung.

[37] Santhanakrishnan R, Wang N, Larson MG, Magnani JW, McManus DD, Lubitz SA (2016). Atr Fibrillation Begets Heart Fail Vice Versa Circulation.

[38] Wang TJ, Larson MG, Levy D, Vasan RS, Leip EP, Wolf PA (2003). Temporal relations of Atrial Fibrillation and Congestive Heart Failure and their joint influence on Mortality. Circulation.

[39] Redfield MM, Kay GN, Jenkins LS, Mianulli M, Jensen DN, Ellenbogen KA. Tachycardia-related cardiomyopathy: a common cause of ventricular dysfunction in patients with atrial fibrillation referred for atrioventricular ablation. Mayo Clin Proc. 2000;75(8):790–5. 10.4065/75.8.790 10943231

[40] Jaïs P, Cauchemez B, Macle L, Daoud E, Khairy P, Subbiah R (2008). Catheter ablation Versus Antiarrhythmic Drugs for Atrial Fibrillation. Circulation.

[41] Wilber DJ, Pappone C, Neuzil P, De Paola A, Marchlinski F, Natale A (2010). Comparison of Antiarrhythmic Drug Therapy and Radiofrequency catheter ablation in patients with Paroxysmal Atrial Fibrillation. JAMA.

[42] Marrouche NF, Brachmann J, Andresen D, Siebels J, Boersma L, Jordaens L (2018). Catheter ablation for Atrial Fibrillation with Heart Failure. N Engl J Med.

[43] Di Biase L, Mohanty P, Mohanty S, Santangeli P, Trivedi C, Lakkireddy D (2016). Ablation Versus Amiodarone for treatment of Persistent Atrial Fibrillation in patients with Congestive Heart Failure and an implanted device. Circulation.

[44] Packer DL, Mark DB, Robb RA, Monahan KH, Bahnson TD, Moretz K (2018). Catheter ablation versus Antiarrhythmic Drug Therapy for Atrial Fibrillation (CABANA) Trial: study rationale and design. Am Heart J.

[45] Fukui A, Tanino T, Yamaguchi T, Hirota K, Saito S, Okada N (2020). Catheter ablation of atrial fibrillation reduces Heart Failure rehospitalization in patients with Heart Failure with preserved ejection fraction. J Cardiovasc Electrophysiol.

[46] Rahman A, Hasani A, Moussa O, Kumar S, Jahufar F, Saeed O (2019). Efficacy of catheter ablation of Atrial Fibrillation in Heart Failure with preserved ejection fraction. J Card Fail.

[47] Machino-Ohtsuka T, Seo Y, Ishizu T, Yamamoto M, Hamada-Harimura Y, Machino T (2019). Relationships between maintenance of sinus rhythm and clinical outcomes in patients with Heart Failure with preserved ejection fraction and atrial fibrillation. J Cardiol.

[48] Kelly JP, DeVore AD, Wu J, Hammill BG, Sharma A, Cooper LB (2019). Rhythm Control Versus Rate Control in patients with Atrial Fibrillation and Heart Failure with preserved ejection fraction: insights from get with the guidelines-Heart failure. J Am Heart Assoc.

[49] Ichijo S, Miyazaki S, Kusa S, Nakamura H, Hachiya H, Kajiyama T (2018). Impact of catheter ablation of atrial fibrillation on long-term clinical outcomes in patients with Heart Failure. J Cardiol.

[50] Black-Maier E, Ren X, Steinberg BA, Green CL, Barnett AS, Rosa NS (2018). Catheter ablation of atrial fibrillation in patients with Heart Failure and preserved ejection fraction. Heart Rhythm.

[51] Eitel C, Ince H, Brachmann J, Kuck KH, Willems S, Gerds-Li JH (2019). Atrial fibrillation ablation strategies and outcome in patients with Heart Failure: insights from the German ablation registry. Clin Res Cardiol.

[52] Hayıroğlu Mİ, Altay S (2023). The role of Artificial Intelligence in Coronary Artery Disease and Atrial Fibrillation. Balkan Med J.

[53] Kotecha D, Piccini JP (2015). Atrial fibrillation in Heart Failure: what should we do?. Eur Heart J.

[54] Prabhu S, Taylor AJ, Costello BT, Kaye DM, McLellan AJA, Voskoboinik A (2017). Catheter ablation Versus Medical Rate Control in Atrial Fibrillation and Systolic Dysfunction. J Am Coll Cardiol.

[55] Quiñones MA, Douglas PS, Foster E, Gorcsan J, Lewis JF, Pearlman AS (2003). American College of Cardiology/American Heart Association clinical competence Statement on Echocardiography. Circulation.

[56] Wu G, Huang H, Cai L, Yang Y, Liu X, Yu B (2021). Long-term observation of catheter ablation vs. pharmacotherapy in the management of persistent and long-standing persistent atrial fibrillation (CAPA study). EP Europace.

[57] Calkins H, Hindricks G, Cappato R, Kim YH, Saad EB, Aguinaga L (2017). 2017 HRS/EHRA/ECAS/APHRS/SOLAECE expert consensus statement on catheter and surgical ablation of atrial fibrillation. Heart Rhythm.

[58] Verma A, Ha ACT, Kirchhof P, Hindricks G, Healey JS, Hill MD (2018). The optimal anti-coagulation for enhanced-risk patients post–catheter ablation for Atrial Fibrillation (OCEAN) trial. Am Heart J.

[59] Suman-Horduna I, Roy D, Frasure-Smith N, Talajic M, Lespérance F, Blondeau L (2013). Quality of life and functional capacity in patients with Atrial Fibrillation and Congestive Heart Failure. J Am Coll Cardiol.

[60] Reynolds MR, Zimetbaum P, Josephson ME, Ellis E, Danilov T, Cohen DJ (2009). Cost-effectiveness of Radiofrequency catheter ablation compared with Antiarrhythmic Drug Therapy for Paroxysmal Atrial Fibrillation. Circ Arrhythm Electrophysiol.

[61] KHAYKIN Y, WANG X, WAZNI NATALEA, SKANES OM, HUMPHRIES AC (2009). Cost Comparison of Ablation Versus Antiarrhythmic Drugs as First-Line Therapy for Atrial Fibrillation: an economic evaluation of the RAAFT Pilot Study. J Cardiovasc Electrophysiol.

[62] Arora S, Jaswaney R, Jani C, Zuzek Z, Thakkar S, Patel HP (2020). Catheter ablation for Atrial Fibrillation in patients with Concurrent Heart Failure. Am J Cardiol.

[63] Gupta A, Perera T, Ganesan A, Sullivan T, Lau DH, Roberts-Thomson KC (2013). Complications of catheter ablation of Atrial Fibrillation. Circ Arrhythm Electrophysiol.

[64] Natale A, Mohanty S, Goldstein L, Gomez T, Hunter TD (2021). Real-world safety of catheter ablation for atrial fibrillation with contact force or cryoballoon ablation. J Interventional Cardiac Electrophysiol.

[65] Shroff N, Choi W, Villanueva-Meyer J, Palacio DM, Bhargava P (2021). Pulmonary vein occlusion: a delayed complication following radiofrequency ablation for atrial fibrillation. Radiol Case Rep.

[66] Chen LY, Chung MK, Allen LA, Ezekowitz M, Furie KL, McCabe P et al. Atrial Fibrillation Burden: moving Beyond Atrial Fibrillation as a Binary Entity: A Scientific Statement from the American Heart Association. Circulation. 2018;137(20). 10.1161/CIR.0000000000000568 PMC8463258 29661944

[67] Pathak RK, Middeldorp ME, Lau DH, Mehta AB, Mahajan R, Twomey D (2014). Aggressive risk factor reduction study for Atrial Fibrillation and implications for the outcome of ablation. J Am Coll Cardiol.

[68] Nesapiragasan V, Hayıroğlu Mİ, Sciacca V, Sommer P, Sohns C, Fink T (2023). Catheter ablation approaches for the Treatment of Arrhythmia Recurrence in patients with a durable pulmonary vein isolation. Balkan Med J.

