# Supplementary material for: Catheter ablation versus medical therapy in atrial fibrillation: an umbrella review of meta-analyses of randomized clinical trials
Source: BMC Cardiovasc Disord. 2024 Feb 29;24:131. doi: 10.1186/s12872-023-03670-5 (PMC10902941; doi:10.1186/s12872-023-03670-5)
Supplement: Supplementary file 2 — Supplementary Material 2: AMSTAR 2: Critical appraisal tool for systematic reviews that includes randomized or non-randomized studies of healthcare interventions, or both [file 12872_2023_3670_MOESM2_ESM.docx]

**Supplemental Table 1. Summary of studies**

| **Source** | **Health outcome** | **Population** | **Intervention (I)** | **Comparison (C)** | **Follow-up (range)** | **No of studies** | **Total Participants** | **Metric** | **ES (95% CI)** | **P Value** | **I^2^ (%)** | **GRADE Overall certainty of evidence** | **AMSTAR 2** |
| --- | --- | --- | --- | --- | --- | --- | --- | --- | --- | --- | --- | --- | --- |
| Barra 2018 [28] | All-cause mortality | Patients with HF | Catheter Ablation | Medical Treatment |  | 10 | 1769 | RR^[[1]](#footnote-1)^ | 0.55 (0.39-0.79) | <0.001 | 0 | High | High |
| Mao 2020 [32] | All-cause mortality | Patients without HF | Catheter Ablation | Medical Treatment |  | 10 | 3479 | RR | 0.9 (0.68-1.2) | 0.48 | 0 | High | High |
| Mao 2020 [32] | All-cause mortality | Patients with and without HF | Catheter Ablation | Medical Treatment |  | 15 | 4685 | RR | 0.72 (0.57-0.9) | 0.005 | 0 | Moderate | High |
| Zheng 2021 [37] | All-cause mortality | HF unspecified | Catheter Ablation | Medical Treatment |  | 12 | 4492 | RR | 0.72 (0.58-0.9) | 0.745 | 0 | Moderate | Critically low |
| Saylik 2023  [39] | All-cause mortality | Patients with HF | Catheter Ablation | Medical Treatment |  | 19 | 2,185 | RR | 0.64 (0.5-0.82) | <0.01 | 0 | High | High |
| Zhu 2021 [38] | Improved LVEF | Patients with HF | Catheter Ablation | Medical Treatment |  | 8 | 947 | MD^[[2]](#footnote-2)^ | 6.45 (3.49-9.41) | <0.0001 | 90 | Very Low | Moderate |
| Song 2022 [35] | Improved LVEF | HF unspecified | Catheter Ablation | Medical Treatment |  | 7 | 958 | WMD^[[3]](#footnote-3)^ | 5.39 (2.45-5.4) | <0.001 | 84.4 | Low | Low |
| Lin 2023  [40] | Improved LVEF | Patients with HF | Catheter Ablation | Medical Treatment |  | 8 | 1,868 | MD | 5.65 (3.32-7.98) | <0.0001 | 86 | Moderate | High |
| Zhu 2021 [38] | Change in MLHFQ | Patients with HF | Catheter Ablation | Medical Treatment |  | 5 | 483 | MD | 8.19 (0.3-16.08) | 0.04 | 90 | Very Low | Moderate |
| Shi 2015 [34] | Change in MLHFQ | Patients with and without HF | Catheter Ablation | Medical Treatment |  | 4 | 737 | WMD | 2.23 (0.24-4.21) | 0.03 | 57 | Low | High |
| Agasthi 2019 [25] | Change in MLHFQ | HF unspecified | Catheter Ablation | Medical Treatment |  | 5 | 424 | MD | 12.14 (2.71-21.56) | 0.05 | 74 | Low | High |
| Kheiri 2018 [30] | Cardiovascular hospitalization | Patients with HF | Catheter Ablation | Medical Treatment |  | 6 | 803 | RR | 0.57 (0.45-0.72) | <0.01 | 0 | High | Low |
| Khan 2020 [33] | Cardiovascular hospitalization | Patients without HF | Catheter Ablation | Medical Treatment |  | 6 | 2988 | RR | 0.37 (0.18-0.77) | 0.01 | 86 | Low | Moderate |
| Mao 2020 [32] | Cardiovascular hospitalization | Patients with and without HF | Catheter Ablation | Medical Treatment |  | 11 | 3589 | RR | 0.61 (0.46-0.81) | 0.0006 | 77 | Low | High |
| Asad 2019 [27] | AF Recurrence | Patients with HF | Catheter Ablation | Medical Treatment |  | 7 | 964 | RR | 0.4 (0.26-0.6) | <0.0001 | 84 | Low | Moderate |
| Asad 2019 [27] | AF Recurrence | Patients without HF | Catheter Ablation | Medical Treatment |  | 13 | 2830 | RR | 0.46 (0.35-0.6) | <0.00001 | 86 | Low | Moderate |
| Asad 2019 [27] | AF Recurrence | Patients with and without HF | Catheter Ablation | Medical Treatment |  | 18 | 3500 | RR | 0.42 (0.33-0.53) | <0.00001 | 87 | Low | Moderate |
| Lui 2018 [31] | AF Recurrence | HF unspecified | Catheter Ablation | Medical Treatment |  | 11 | 1558 | RR | 0.46 (0.37-0.57) | <0.001 | 72.2 | Moderate | High |
| Lin 2023  [40] | AF Recurrence | Patients with HF | Catheter Ablation | Medical Treatment |  | 8 | 2059 | OR | 0.23 (0.11-0.48) | <0.00001 | 82 | Very low | High |
| Barra 2018 [28] | Cardiovascular event | Patients with HF | Catheter Ablation | Medical Treatment |  | 7 | 1289 | RR | 0.94 (0.46-1.94) | 0.87 | 0 | High | High |
| Khan 2020 [33] | Cardiovascular event | Patients without HF | Catheter Ablation | Medical Treatment |  | 3 | 2496 | RR | 0.55 (0.18-1.66) | 0.29 | 0 | Moderate | Moderate |
| Mao 2020 [32] | Cardiovascular event | Patients with and without HF | Catheter Ablation | Medical Treatment |  | 9 | 3849 | RR | 0.7 (0.39-1.23) | 0.21 | 0 | High | High |
| Song 2022 [35] | Cardiovascular event | Patients with HF | Catheter Ablation | Medical Treatment |  | 11 | 4620 | RR | 0.61 (0.39-0.96) | 0.035 | 0 | Moderate | Low |
| Androulakis 2022 [26] | Major bleeds | Patients with HF | Catheter Ablation | Medical Treatment |  | 4 | -- | RR | 0.005 (0.001-0.013) | 0.777 | 0 | Low | High |
| Mao 2020 [32] | Major bleeds | Patients with and without HF | Catheter Ablation | Medical Treatment |  | 10 | 1411 | RR | 3.88 (1.63-9.22) | 0.002 | 0 | Moderate | High |
| Zheng 2021 [37] | Major bleeds | HF unspecified | Catheter Ablation | Medical Treatment |  | 11 | 4066 | RR | 1.09 (0.75-1.58) | 0.431 | 1 | High | Critically low |
| Mao 2020 [32] | Pulmonary vein stenosis | Patients with and without HF | Catheter Ablation | Medical Treatment |  | 10 | 3839 | RR | 3.94 (1.49-10.38) | 0.006 | 0 | High | High |
| Zheng 2021 [37] | Pulmonary vein stenosis | HF unspecified | Catheter Ablation | Medical Treatment |  | 7 | 3531 | RR | 2.34 (0.78-7.07) | 0.935 | 0 | High | Critically low |
| Mao 2020 [32] | Recurrence of any atrial arrhythmia | Patients with and without HF | Catheter Ablation | Medical Treatment |  | 26 | 4782 | RR | 0.43 (0.37-0.51) | <0.00001 | 81 | Low | High |
| Chen 2020 [29] | Major adverse events | Patients with HF | Catheter Ablation | Medical Treatment |  | 7 | 1112 | OR | 0.66 (0.33-1.34) | 0.25 | 64 | Moderate | High |
| Yi 2019 [36] | Major adverse events | HF unspecified | Catheter Ablation | Medical Treatment |  | 8 | 1246 | OR | 1.29 (0.88-1.88) | 0.2 | 16 | High | Low |
| Lin 2023  [40] | Major adverse events | Patients with HF | Catheter Ablation | Medical Treatment |  | 8 | 2,155 | OR | 1.06 (0.83-1.35) | 0.66 | 48 | Moderate | High |
| Razzak 2022  [41] | Major adverse events | Patients with and without HF | Catheter Ablation | Medical Treatment |  | 6 | 1,212 | RR | 0.9 (0.56-1.44) | 0.65 | 43 | Moderate | High |

1. RR = Relative Risk [↑](#footnote-ref-1)
2. MD = Mean difference [↑](#footnote-ref-2)
3. WMD = Weighted Mean Difference [↑](#footnote-ref-3)
